# Human pegivirus (HPgV, GBV-C) RNA in volunteer blood donors from a public hemotherapy service in Northern Brazil

**DOI:** 10.1186/s12985-020-01427-6

**Published:** 2020-10-14

**Authors:** Aniel de Sarom Negrão Silva, Clayton Pereira Silva, Rafael Ribeiro Barata, Pedro Victor Reis da Silva, Patrícia Danin Jordão Monteiro, Letícia Lamarão, Rommel Mário Rodríguez Burbano, Márcio Roberto Teixeira Nunes, Patrícia Danielle Lima de Lima

**Affiliations:** 1 Center for Life Science and Health, Pará State University, Travessa. Perebebuí, 2623, Marco, Belém, Pará 66087-662 Brazil; 2 Foundation Center for Hemotherapy and Hematology of Pará (HEMOPA Foundation), Travessa Padre Eutíquio, 2109, Batista Campos, Belém, Pará 66033-000 Brazil; 3 Evandro Chagas Institute, Rodovia BR-316, km 7 s/n, Levilândia, Ananindeua , Pará 67030-000 Brazil; 4 Ophir Loyola Hospital, Av. Governador Magalhães Barata, 992, São Brás, Belém , Pará 66063-240 Brazil

**Keywords:** Pegivirus, Blood donors, Prevalence, Genome, HIV coinfection

## Abstract

**Background:**

Human pegivirus (HPgV)—formerly known as GBV-C—is a member of the *Flaviviridae* family and belongs to the species *Pegivirus* C. It is a non-pathogenic virus and is transmitted among humans mainly through the exposure to contaminated blood and is often associated with human immunodeficiency virus (HIV) infection, among other viruses. This study aimed to determine the prevalence of HPgV viremia, its association with HIV and clinical epidemiological factors, as well as the full-length sequencing and genome characterization of HPgV recovered from blood donors of the HEMOPA Foundation in Belém-PA-Brazil.

**Methods:**

Plasma samples were obtained from 459 donors, tested for the presence of HPgV RNA by the RT-qPCR. From these, a total of 26 RT-qPCR positive samples were submitted to the NGS sequencing approach in order to obtain the full genome. Genome characterization and phylogenetic analysis were conducted.

**Results:**

The prevalence of HPgV was 12.42%. We observed the highest prevalences among donors aged between 18 and 30 years old (16.5%), with brown skin color (13.2%) and men (15.8%). The newly diagnosed HIV-1 prevalence was 26.67%. The HPgV genotype 2 (2a and 2b) was identified. No data on viral load value was found to corroborate the protective effect of HPgV on HIV evolution.

**Conclusions:**

This study provided information regarding the HPgV infection among blood donors from HEMOPA Foundation. Furthermore, we genetically characterized the HPgV circulating strains and described by the first time nearly complete genomes of genotype 2 in Brazilian Amazon.

## Background

Human pegivirus (HPgV), formerly known as GBV-C or hepatitis G virus (HGV), is a member of the *Flaviviridae* family, belongs to the species *Pegivirus* C [1]. HPgV is an enveloped virus with a single-stranded, positively polarized RNA genome comprising approximately 9,400 nucleotides. The viral genome is similar to the genome of the hepatitis C virus and contains a single open reading frame (ORF) located between the untranslated regions (UTRs) at the 5′ and 3′ ends of the viral genome. The 5′-NTR region is highly conserved with an internal ribosome entry site (IRES) and is responsible for the initiation of the translation of the viral RNA, resulting in the synthesis of a polyprotein of approximately 3,000 amino acid residues. Through the action of cellular peptidases and viral proteases, the polyprotein is cleaved to produce eight mature yet incompletely characterized proteins, including the two structural (E1 and E2) and seven non-structural (NS) proteins [2–4].

HPgV is transmitted among humans mainly through exposure to contaminated blood. This transmission profile deems HPgV as a common coinfection with other viruses such as HIV-1, hepatitis C virus (HCV), and Ebola virus [5–7]. Up to 40% of the individuals infected with HIV and/or HCV are positive for HPgV infection [8, 9]

People HIV-1 co-infected with HPgV experience slower disease progression that may be influenced by the interference of HPgV on the pathogenicity of HIV-1 [10, 11]. However, the mechanism by which HPgV mediates this protective effect remains inconclusive [12, 13].

Several studies carried out in different populations in the last decades in Brazil have shown varying prevalence rates of HPgV infections [14, 15]. Studies among healthy blood donors conducted in Brazil revealed prevalence rates of 19.5% and 9.7% among individuals with prior exposure and active infection, respectively. [16]. However, the most significant prevalence reported was among patients with HIV, with a value reaching up to 34% [17].

The prevalence of the virus is lower in the developed countries (1–5%) than in the developing countries -(approximately 20%), with South America exhibiting a prevalence rate of up to 14.6% among blood donors [4]. Seroprevalence studies revealed the presence of anti-E2 antibodies in 19.5% of healthy blood donors [18]. However, data about the soroprevalence of HPgV viremia and its circulating strains in the Northern Brazilian population are scarce, particularly among blood donors.

This study aimed to determine the prevalence of HPgV viremia and its association with clinical epidemiological factors and the presence of HIV, as well as the complete genome characterization of HPgV strains in volunteer blood donors from a public hemotherapy service in Northern Brazil.

## Methods

### Blood donors and the collection of serum samples

A cross-sectional study was performed to determine the prevalence of HPgV infection among blood donors from the HEMOPA Foundation between March 2017 and April 2018. Epidemiological data were obtained through access to the HEMOPA Foundation donor registry. The sample size was calculated using EpiInfo™ software [19] based on the presumed prevalence of 5% to 10% of HPgV in Brazil [17, 20]. For this calculation, the number of blood donors registered in 2016 at the HEMOPA Foundation (63,501), 95% confidence level, and 20% margin adjustment was used to obtain a total of 366 individuals. A total of 459 serum samples (400 μL) from the blood donors from the HEMOPA Foundation were tested.

### Extraction and detection of HIV, HCV, and HPgV nucleic acids

The extraction of nucleic acids was performed using the QIAmp RNA mini Kit (Qiagen®, Hilden, Germany) according to the manufacturer’s recommendations. HIV and HCV detection were performed with Hemocenter’s Nuclear Acid Test Platform (NAT) using the HIV/HCV NAT kit (Bio Manguinhos®, Rio de Janeiro, Brazil), according to the manufacturer’s recommendations.

The presence of HPgV nucleic acid was evaluated by the RT-qPCR, using the custom Assay TaqMan® Fast Virus 1-Step, developed by AB Applied Biosystems (Foster City, California, EUA), following the manufacturer’s Fast protocol as follows: 1 cycle of reverse transcription (RT) for 2 min at 50 °C; inactivation of Reverse Transcription (RT)/start of denaturation (1 cycle) for 20 s at 95 °C; amplification for 40 cycles of 95 °C for 3 s and 60 °C for 30 s. The selected primers corresponded to the 5′-UTR of the viral genome according to GenBank NC_001710 and were as follows: RTG1 (GTGGTGGATGGGTGATGACA; sense), RTG2 (GACCCACCTATAGTGGCTACCA; antisense), and NFQ (5′-FAM-CCGGGATTTACGACCTACC3′; probe) [17].

### Quantification of HIV-1 and HPgV plasma viral load

HIV-1 viral load was measured in a Real-Time Rotor-Gene® Q platform using *artus* HI Virus-1 RG RT-PCR (QIAGEN Hilden, Germany) and HPgV viral load was measured in a Real-Time LightCycler® 480 Instrument II (Roche Applied Science, Penzberg, Germany) using TaqMan® Fast Virus 1-Step Master Mix (Foster City, California, USA). Both methods strictly followed the manufacture´s recommendation.

### High-throughput sequencing

The RNA, obtained in the nucleic acid extraction step, was quantified in Qubit 2.0 fluorometer (Thermo Fisher Scientific), using the QubitTM RNA HS Assay Kit 500 assays (Invitrogen by Thermo Fisher Scientific). Then, cDNA was synthesized using the cDNA Synthesis System Roche® kit (Roche Applied Science), as described by the manufacturer. The subsequent step was the quantification of cDNA using the Qubit 2.0 fluorometer (Thermo Fisher Scientific), using the QubitTM dsDNA HS Assay Kit (Invitrogen by Thermo Fisher Scientific) and analysis of cDNA integrity in the equipment 2100 Bioanalyzer (Agilent Technologies) using the high sensitivity DNA reagents kit (Agilent Technologies). Genome sequencing was performed using the HiSeq 2500 platform (Illumina) as previously described [21].

### Bioinformatics analysis

Generated reads were filtered, adapters and reads with Phred quality scores below 20 and size less than 50 nt, were removed using Trim Galore 0.4.4, Cutadapt and Prinseq-lite 0.20.4 software [22–24]. The filtered reads were used in de novo assembly strategy applying two software: IDBA-UD v.1.1.3 [25] and MEGAHIT v.1.1.3 [26], both set to a *k-mer* range of 21 to 91, varying every *10 k-mer.* For the removal of redundant data, generated contigs were processed using CD-Hit-Est v.4.7 [27] set to a threshold of 90% identity. Then, the non-redundant contigs were aligned against the NCBI non-redundant protein database using the Blastx (https://blast.ncbi.nlm.nih.gov/Blast.cgi?PROGRAM=blastx&PAGE_TYPE=BlastSearch&LINK_LOC=blasthome) algorithm and the software Diamond v.0.9.22 [28].

### Genome characterization and phylogenetic inference

The HPgV genomes sequences, identified by the Blastx algorithm, were used for predicting the coding region (Open Reading Frame; ORF), as well as the 5′-UTR and 3′-UTR regions using the Geneious v9 tool. Viral genomes were aligned with other HPgV complete genomes available in GenBank database using the MAFFT v7 software [29]. For phylogenetic analyses, complete ORFs from aligned genomes were used to perform the phylogenetic reconstructions using the maximum-likelihood method, generated by RAxML v.8.2.12 [30], applying 1000 bootstrap replicates [31] and the best nucleotide replacement model calculated by JModelTest [32]. Complete genome sequences of HPgV obtained in this study have been submitted to GenBank (accession numbers MN215894–MN21591).

### Statistical analysis

The differences between the groups were analyzed with the chi-square test, G test of independence, Student’s *t*-test, and odds ratio. The level of significance of α = 0.05 was adopted for the rejection of the null hypothesis. Statistical analyses were performed using the BioEstat program version 5.3 and GraphPad Prism version 8; Microsoft Excel Professional 2007 program was used for data processing and to prepare tables and databases.

## Results

Plasma samples were obtained from 459 donors at the time of screening. The prevalence of HPgV in the samples was 12.42% (n = 57) and a total of 26.14% (n = 120) of the donors were HIV positive, diagnosed through routine tests carried out at the HEMOPA Foundation. The HPgV prevalence among HIV donors was 26.67% (n = 32, p < 0 0.0001). HIV positive donors were four times more likely to have HPgV infection than those HIV negative (odds ratio = 4.56, p < 0.0001, 95% confidence interval [CI]:2.57–8.10).

The highest prevalences of HPgV were observed among donors with ages ranging from 18 to 30 years old (16.5%, p = 0.024), men (15,8%, p = 0.005) with brown skin color (13.2%, p = 0,462) and 12 or more years of education (24.0%, p < 0.0001) (Table 1).

**Table 1:** Clinical epidemiological data on the prevalence of HPgV among blood donors

Variable/category	Total		HPgV^+^		HPgV^−^		p-value
	n	(%)	n	(%)	n	(%)	
*Age (years)*							
18–30	224	48.8	37	16.5	187	83.5	p = 0.0273
31–50	197	42.9	18	9.1	179	90.9	
≥ 51	38	8.3	2	5.3	36	94.7	
*Skin color*							
Brown	356	77.6	47	13.2	309	86.8	p = 0.462
White	84	18.3	9	10.7	75	89.3	
Black	19	4.1	1	5.3	18	94.7	
*Sex*							
Male	284	61.9	45	15.8	241	84.9	p = 0.0056
Female	175	38.1	12	6.9	161	92.0	
*Education (years of study)*							
≥ 12	146	31.8	35	24.0	111	76.0	p < 0.0001
9 to 11	259	56.4	19	7.3	240	92.7	
≤ 8	54	11.8	3	5.6	51	94.4	
*Co-infection*							
HIV+	120	26.1	32	26.7	88	73.3	p < 0.0001
HIV−	339	73.9	25	7.4	314	92.6	

In 18 of the 57 HPgV positive samples (31.6%) near-complete genomes sequences were obtained. The BlastX result of the 18 genomes obtained showed 91% to 93.24% identity with Human pegivirus sequences from the United Kingdom (LT009489 and LT009494), France (MH053115) and Japan (D87255), available from GenBank / NCBI (Table 2). The pairwise alignment of the polyprotein amino acid sequences of these four strains of the bank along with the 18 described sequences showed an identity of 98.6%.

**Table 2:** BlastX result for 18 nearly complete HPgV genome sequences obtained from blood donors from Belém-PA-Brazil

Sample	Genome lenth	Mean cover	Best hit	Query cover (%)	E-value	Identity (%)	Accession
P01	8,392	14,8	Human pegivirus isolate 56,330,229	100	0	92.14	LT009489
P02	8,995	20,1	Human pegivirus isolate JD2B2C	99	0	91.00	MH053115
P09	8,933	22,9	Human pegivirus isolate 56,330,229	99	0	92.34	LT009489
P13	9,101	593,8	Human pegivirus isolate JD2B2C	100	0	91.63	MH053115
P21	9,172	448,6	Hepatitis G virus	100	0	93.24	D87255
P22	8,811	26	Human pegivirus isolate JD2B2C	99	0	91.60	MH053115
P23	9,190	133,7	Human pegivirus isolate JD2B2C	99	0	91.73	MH053115
P24	9,306	386	Human pegivirus isolate 56,330,229	99	0	92.80	LT009489
P25	9,241	327,1	Human pegivirus isolate JD2B2C	99	0	91.64	MH053115
P26	9,189	143,7	Human pegivirus isolate JD2B2C	99	0	91.92	MH053115
P27	8,873	22,6	Human pegivirus isolate 56,330,229	99	0	92.29	LT009489
P28	8,913	32,5	Hepatitis G virus	99	0	93.21	D87255
P31	9,521	538,5	Human pegivirus isolate 56,330,229	99	0	92.51	LT009489
P32	9,256	182,7	Human pegivirus isolate 56,330,286	99	0	92.25	LT009494
P33	9,409	640	Human pegivirus isolate 56,330,286	100	0	92.40	LT009494
P34	9,270	146,1	Human pegivirus isolate 56,330,229	100	0	92.44	LT009489
P35	9,198	755,6	Human pegivirus isolate 56,330,229	100	0	92.22	LT009489
P55	9,203	566,4	Human pegivirus isolate 56,330,229	100	0	92.27	LT009489

**Fig. 1:**
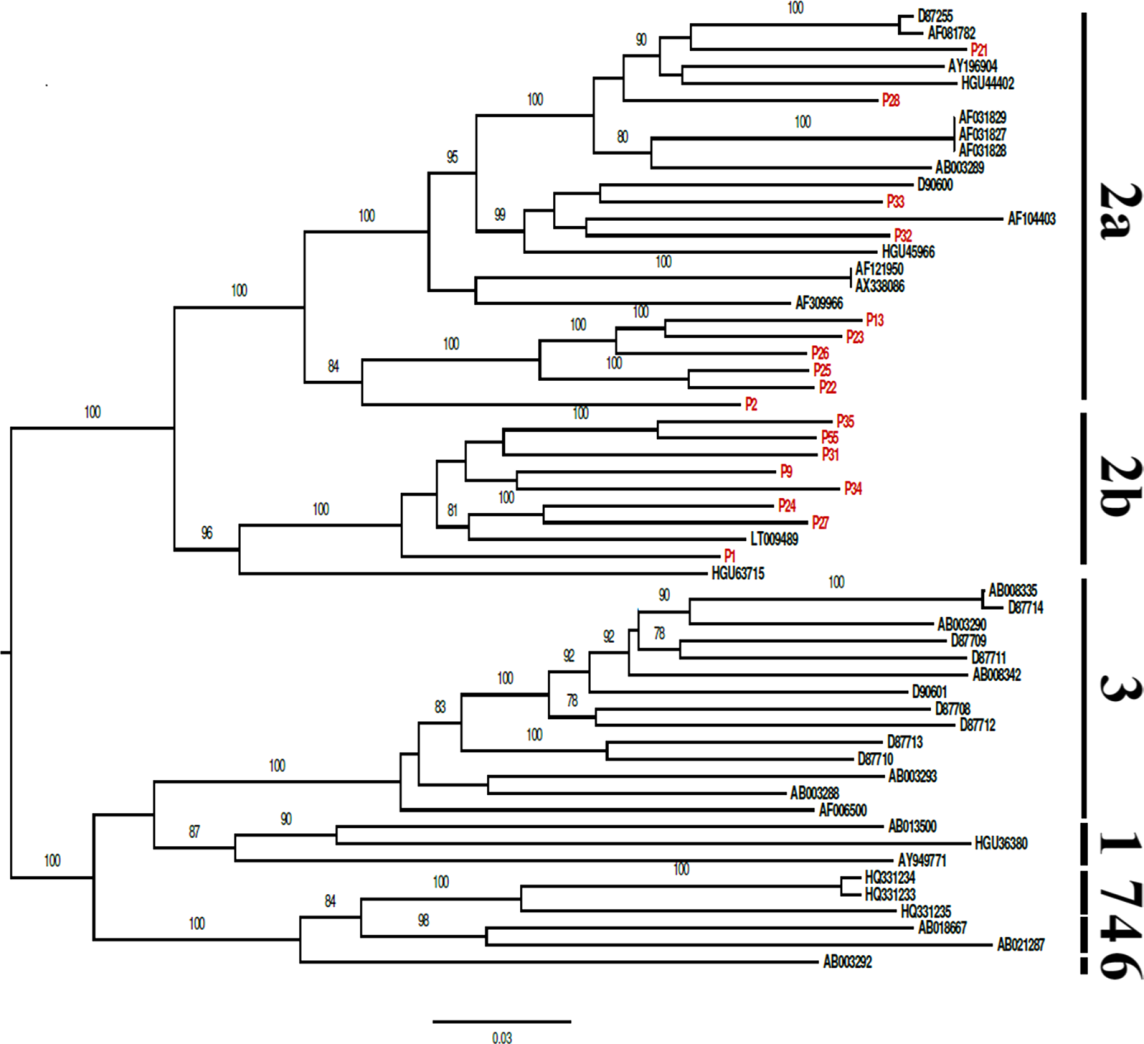
Phylogenetic tree of Human pegivirus (HPgV) generated with complete polyprotein ORF, using RAxML with the GTR + I + G + F nucleotide substitution model using 1000 bootstrap replicas displaying only values greater than 70

All nearly complete genome sequences showed the common genome organization related to members of the HPgV: unique and large ORF flanked by 5′and 3′ UTRs. The phylogenetic tree represented the genotypes (1, 2a, 2b, 3, 4, 6 and 7), including two subgenotypes (2a and 2b). All the sequences of the HPgV genome obtained clustered with sequences of the HPgV genotype 2, subgenotypes 2a and 2b, with high bootstrap values (> 90%) (Fig. 1).

**Fig. 2:**
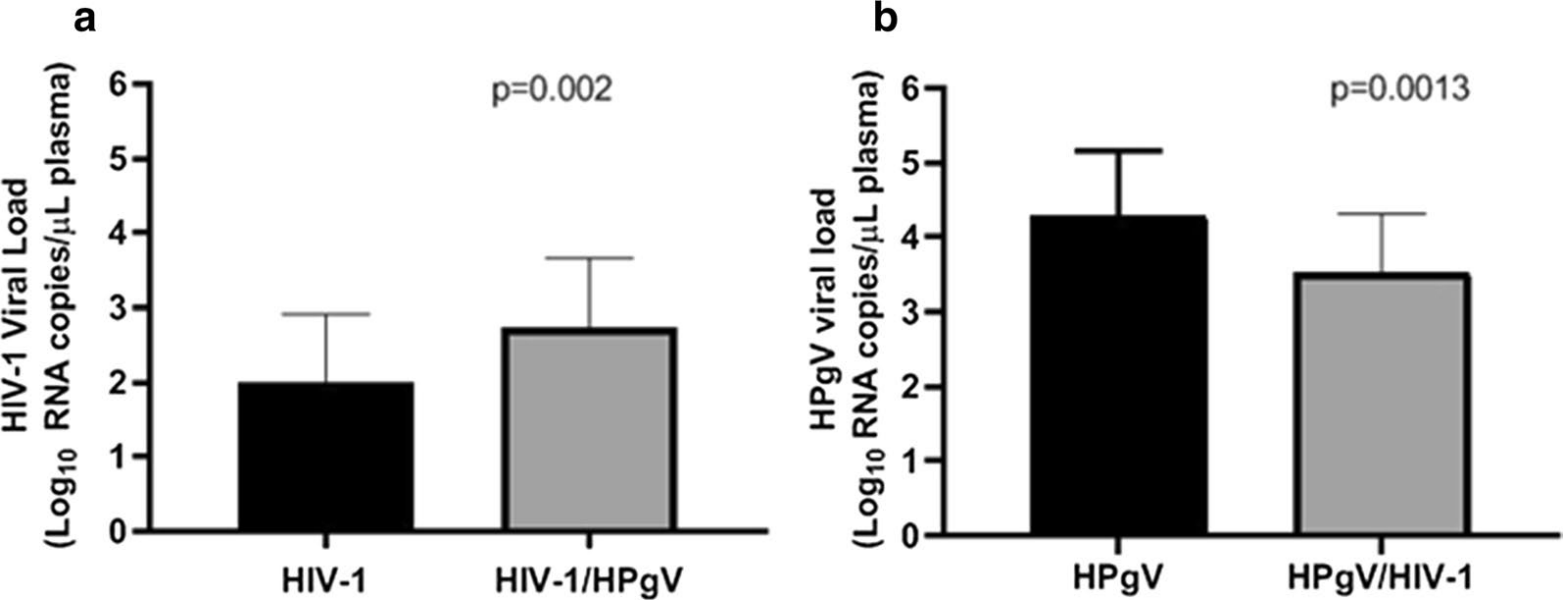
Virological profiles of newly diagnosed blood donors with HIV-1 and HPgV. **a** Comparison of plasma HIV-1 viral load between HIV-1 monoinfected group and HIV-1/HPgV coinfected group. **b** Comparison of plasma HPgV viral load between HPgV monoinfected group and HPgV/HIV-1 coinfected group

Viral load was compared in two groups. First we compared HIV viral load in monoinfected (HIV-1) and coinfected (HIV-1/HPgV). Then, we compared HPgV viral load in monoinfected (HPgV) and coinfected group (HPgV/HIV-1). We found a higher HIV-1 viral load in the coinfected (2.72 Log_10_) than in the monoinfected group (2.00 Log_10;_ Fig. 2a). While a higher HPgV viral load (4.28 Log_10_) was observed in the monoinfected group in comparison with coinfected group (HPgV, Fig. 2b).

## Discussion

The prevalence of HPgV-1 among blood donors was 12,4%, which is consistent and not significantly different from the expected prevalence in developing countries (up to 20%) [4, 33]. The prevalence calculated in this study was 2,8% higher than reported Slavov et al. [34] in a study among blood donors from the city of Macapá (northern Brazil). Previous studies have shown that the prevalence of HPgV among blood donors in most regions of Brazil varies from 5 to 10% [16, 35–38], although Da Mota et al. [39] have found a high prevalence of 21.7% in the southernmost region of Brazil.

In our findings, the highest prevalence of HPgV occurred among subjects between 18 and 30 years of age (16.5%), males (15.8%), and brown individuals (13.2%). It is important to highlight that the epidemiological profile of the donors was similar to that observed in the epidemiology of HIV/AIDS in Brazil, wherein the majority of the infected individuals were male and young subjects (15 to 39 years) with up to 11 years of study (completed high school) [40].

The prevalence of HPgV among the individuals diagnosed with HIV-1 reported in this study was 26,7%, which is 9,7% higher than that reported by Miranda et al. [41]. [38] The high prevalence of HPgV among HIV-1 individuals has been reported in several studies in Brazil and the world [42–44]. The association between the presence of HPgV and HIV is owing to the fact that HPgV likely acts as a protective factor for the development of HIV [44–46].

As was seen in some studies, HIV-1 infected people have reduced mortality when co-infected with HPgV [47, 48]. This protective effect may be due to the inducing effect of HPgV on CD4 and CD8 T lymphocytes, leading to antiretroviral factors secretion and also to the reduction of the expression of the HIV-1 co-receptor CCR5, as reported by Jung et al. and Xiang et al., on in vitro experiments [49, 50]. Nevertheless, the present study showed no evidence of viral load value that corroborated with the protective effect of HPgV in the evolution of HIV-1, instead, HIV-1 viral load in the coinfected group (HIV-1/HPgV) was 0.72 Log_10_ (p = 0,002) higher than in a monoinfected group (HIV-1 positive). Another consideration is that all individuals in our sample were newly diagnosed with HIV-1 during the acute phase, suggesting that HPgV does not exert a protective effect on the pathogenesis of HIV-1 during the acute phase as suggested Bailey et al. [51].

On trials investigating the interaction of SPgV (Simian Pegivirus) and SIV (Simian immunodeficiency virus) infection, Bailey et al. found no evidence of a protective effect of SPgV on the evolution of SIV in the acute phase of infection. The protective immunomodulatory effect of SPgV was observed only in the chronic phase of SIV infection [51]. Extending this observation, our findings corroborate the hypothesis suggested that HPgV does not exert a protective effect during the acute phase of HIV infection, since the HIV positive individuals in this study were all newly diagnosed. Otherwise, as seen in several other studies, there is a likely beneficial relationship between HPgV and the chronic phase of HIV infection [4, 47, 52, 53].

The phylogenetic analysis revealed the presence of genotype 2 and the subtypes 2a and 2b in the studied population. These findings corroborate previous studies that identified these same genotypes in other regions of Brazil [20, 35, 42] and in Brazilian Amazon [34].

HPgV is known as a non-pathogenic virus and is not part of the routine diagnosis in the HEMOPA Foundation, but further studies are necessary to evaluate the unclear aspects related to HPgV infection especially those related to viral biology and interaction with HIV-1. This study genetically characterized and identified, by the first time, the circulating strains of HPgV among blood donors from HEMOPA Foundation and described by the first time nearly complete genomes of genotype 2 in Brazilian Amazon.

## Conclusions

This study provided information regarding the HPgV infection among blood donors from HEMOPA Foundation. Furthermore, we genetically characterized the HPgV circulating strains and described by the first time the genotype 2 genomes in the Brazilian Amazon region.

## Data Availability

Not applicable.

## Abbreviations

cDNA: Complementary deoxyribonucleic acid
dsDNA: Double-stranded deoxyribonucleic acid
GBV-C: GB virus C
HCV: Hepatitis C virus
HEMOPA Foundation: Foundation Center for Hemotherapy and Hematology of Pará
HIV: Human immunodeficiency virus
HPgV: Human Pegivirus
IRES: Internal ribosome entry site
ORF: Open reading frame
RNA: Ribonucleic acid
RT-qPCR: Reverse transcription real-time polymerase chain reaction
SIV: Simian immunodeficiency virus.
SPgV: Simian Pegivirus
UTRs: Untranslated regions
